# Automated contouring, treatment planning, and quality assurance for VMAT craniospinal irradiation (VMAT-CSI)

**DOI:** 10.3389/fonc.2024.1378449

**Published:** 2024-04-10

**Authors:** Eric Simiele, Ignacio O. Romero, Jen-Yeu Wang, Yizheng Chen, Yuliia Lozko, Yuliia Severyn, Lawrie Skinner, Yong Yang, Lei Xing, Iris Gibbs, Susan M. Hiniker, Nataliya Kovalchuk

**Affiliations:** Department of Radiation Oncology, Stanford University, Stanford, CA, United States

**Keywords:** auto-planning, craniospinal irradiation (CSI), VMAT-CSI, auto-contouring, Eclipse Scripting API (ESAPI), deep learning

## Abstract

**Purpose:**

Create a comprehensive automated solution for pediatric and adult VMAT-CSI including contouring, planning, and plan check to reduce planning time and improve plan quality.

**Methods:**

Seventy-seven previously treated CSI patients (age, 2-67 years) were used for creation of an auto-contouring model to segment 25 organs at risk (OARs). The auto-contoured OARs were evaluated using the Dice Similarity Coefficient (DSC), 95% Hausdorff Distance (HD95), and a qualitative ranking by one physician and one physicist (scale: 1-acceptable, 2-minor edits, 3-major edits). The auto-planning script was developed using the Varian Eclipse Scripting API and tested with 20 patients previously treated with either low-dose VMAT-CSI (12 Gy) or high-dose VMAT-CSI (36 Gy + 18 Gy boost). Clinically relevant metrics, planning time, and blinded physician review were used to evaluate significance of differences between the auto and manual plans. Finally, the plan preparation for treatment and plan check processes were automated to improve efficiency and safety of VMAT-CSI.

**Results:**

The auto-contours achieved an average DSC of 0.71 ± 0.15, HD95 of 4.81 ± 4.68, and reviewers’ ranking of 1.22 ± 0.39, indicating close to “acceptable-as-is” contours. Compared to the manual CSI plans, the auto-plans for both dose regimens achieved statistically significant reductions in body V50% and D_mean_ for parotids, submandibular, and thyroid glands. The variance in the dosimetric parameters decreased for the auto-plans as compared to the manual plans indicating better plan consistency. From the blinded review, the auto-plans were marked as equivalent or superior to the manual-plans 88.3% of the time. The required time for the auto-contouring and planning was consistently between 1-2 hours compared to an estimated 5-6 hours for manual contouring and planning.

**Conclusions:**

Reductions in contouring and planning time without sacrificing plan quality were obtained using the developed auto-planning process. The auto-planning scripts and documentation will be made freely available to other institutions and clinics.

## 1 Introduction

Craniospinal irradiation (CSI) plays a crucial part in the multidisciplinary care of CNS tumors in children and adults (1–3). Postoperative CSI with chemotherapy is the current standard of care of medulloblastoma (1, 2, 4). Additionally, CSI in conjunction with total body irradiation (TBI) can be used as a conditioning regimen for stem cell transplantation for patients with acute lymphoblastic leukemia (ALL) with CNS involvement (5, 6).

Radiation treatments for CSI have historically involved simple 3D techniques that lead to significant short and long-term toxicities due to the unnecessary dose delivered to uninvolved organs. To reduce these effects, Chen et al. (7) introduced Volumetric Modulated Arc Therapy (VMAT), which is a modern alternative to the classic 3D treatment technique of these cancers. However, due to the extent and shape of the target volume, this approach is resource and time-intensive in terms of target and organs-at-risk (OAR) contouring, treatment planning, and treatment delivery. The European Society for Pediatric Oncology (SIOPE) radiotherapy group developed extensive guidelines for target volume delineation and provided the recommended OAR structures to consider for pediatric and young adult CSI treatments (8). Due to the large number of OARs to contour and the specifications of the target volume, CSI treatment planning can consume many hours from radiation oncologists, dosimetrists, and medical physicists. Furthermore, inter-planner variability can significantly impact the final plan quality of complex treatment techniques, such as VMAT CSI. Therefore, there is a need to standardize and automate VMAT-CSI treatment planning without sacrificing plan quality.

Previous groups have investigated the feasibility of automating the planning process for CSI (9–11). Wang et al. (10) developed an automated planning process for robust IMRT CSI in RayStation (RaySearch Laboratories, Stockholm, Sweden) and shared their script publicly. They found no significant differences between the manual and autoplans for the 10 patients in their study, but observed a decrease in planning time of approximately 50%. Zhang et al. (11) developed an automated planning technique for VMAT-CSI utilizing PlanIQ Feasibility software (Sun Nuclear, Melbourne, FL) integrated into the Pinnacle treatment planning system. They observed significant decreases in the doses to OARs compared to manual planning, but no significant changes compared to a conventional auto-planning technique without PlanIQ. They only observed a minor decrease in planning time of 12 minutes (on average) using their developed technique compared to manual planning. Hernandez et al. (9) developed an automated contouring and planning tool in RayStation for 3D conventional CSI treatments targeted at improving safety and efficiency in low and middle income countries. While these prior works have automated parts of the treatment planning process, no study has automated the entire chain of treatment planning from contouring to physics second check for a modern CSI treatment technique such as VMAT-CSI. Only Wang et al. (10) shared their developed scripts with the public via GitHub, however, many of the parameters including dose levels, structure names, and optimization structures are hard-coded parameters that follow conventions used in their clinic, which may limit widespread adoption of their technique.

This goal of this work was to automate the contouring, treatment planning, and physics plan check process for VMAT-CSI. All developed software in this work will target the Eclipse treatment planning system (Varian Medical Systems Inc., Palo Alto, CA). In addition, the results of this work, including developed software, will be shared publicly to enable institutions to adopt the developed auto-planning process into their own practice. To our knowledge, this is the first work to develop a comprehensive auto-contouring and auto-planning tool for VMAT-CSI.

## 2 Methods

A figure of the workflow for the developed auto-planning process is provided in the **Supplementary Material** (**Supplementary Figure 1**). Each step in the workflow is described in more detail in the following sections. The use of anonymized patient data for this study was approved by the institutional review board.

### 2.1 Auto-contouring

#### 2.1.1 Training and test data

Auto-contouring was implemented for 25 OARs including brain, brainstem, optic chiasm, parotids, heart, lungs, kidneys, etc. A total of 77 previously treated CSI patients (age range, 2 - 67 years, median, 13.3 years) were used for the development of the auto-contouring model and divided into 50 training cases, 7 validation cases, and 20 test cases. For each case, the CT scan and RT structure set used for radiation treatment planning were thoroughly reviewed by two radiation oncologists and two physicists to standardize contours and structure names.

#### 2.1.2 Auto-contouring model architecture and training details

A classic 2D U-Net architecture was selected to perform the auto-segmentation of 20 OARs, and a modified 2.5D U-Net architecture was selected to perform the auto-segmentation of the remaining 5 OARs (optic chiasm, left optic nerve, right optic nerve, left cochlea, and right cochlea). Two deep learning models were employed in this work as it was observed during training and testing that the 2D model performed well for larger structures, but poorly for small structures. Utilizing a 2.5D model significantly increased the accuracy of the segmentation of these smaller structures, but also significantly increased the time required for segmentation, with little gain in accuracy for the larger structures. Therefore, a hybrid approach consisting of two models represented an ideal balance between speed and accuracy of the auto-contouring process.

The input to both networks is a 192×352-pixel image, and the output is a 192×352-pixel binary mask for each of the 25 OARs. Both networks consist of an encoder section composed of four contraction blocks and a decoder section composed of four expansion blocks. Each contraction block consists of two 2D convolution layers followed by 2D maximum pooling and doubles the number of kernels to encode features of greater complexity. At the bottom of the networks, two standalone 2D convolution layers of kernel size 1024 connect the encoder section to the decoder section. Subsequently, each expansion block in the decoder section consists of a 2D transposed convolution operator followed by two 2D convolution layers to perform spatial up-sampling. Skip connections between symmetrical contraction and expansion blocks facilitate the concatenation of lower-level information to processed information in latter layers of the network. At the end of the decoder section, two standalone 2D convolution layers of kernel size 64 are followed by sigmoid activation, classifying each pixel as part of or outside of each of the OARs. In the 2.5D U-Net, three consecutive 2D image slices with an interval of 2, i.e., slices n-2, n, and n+2, are input into the network, and the auto-segmentation predictions of the three slices are output. The 2.5D network is applied for the whole image volume slice by slice, and the predictions for each 2D image slice are staked and averaged as the final auto-segmentation. This 2.5D method considers the continuity and variability of the organ structure, which provided auto-segmentation accuracy superior to that of the 2D method for the 5 OARs.

For training, the hyperparameters were set to 70 epochs, batch size of 8, initial learning rate of 0.001. The Adam optimizer and Dice loss function were used for the network optimization. The model was implemented, trained, and evaluated using Python 3.10 and the CUDA-enabled PyTorch 1.13 library. An Alienware Aurora R15 workstation with 13^th^-generation Intel Core i9 CPU, NVIDIA GeForce RTX 4090 GPU, and 64 GB RAM was used to train the auto-contouring model. The model was deployed for production in the clinical setting on a Dell Precision workstation with Intel Xeon Silver 4110 CPU, NVIDIA Quadro P5000, and 32 GB RAM.

#### 2.1.3 Auto-contouring evaluation metrics

To measure spatial overlap, Dice Similarity Coefficient (DSC) between predicted and ground truth labels was calculated using Equation 1 where A and B are the predicted and ground truth volumes, respectively:


(1) $$DSC=\frac{2\left|A\cap^{\;}B\right|}{\left|A\right|+\left|B\right|},$$

To evaluate surface-to-surface distance, 95th percentile Hausdorff Distance (HD95) between predicted and ground truth labels was calculated using the MedPy 0.4.0 library. A qualitative ranking of auto-contours was also performed by one physician and one physicist using the clinical trials contour ranking scale: 1 - acceptable, 2 - minor edits, and 3 – major edits.

### 2.2 Auto-planning

#### 2.2.1 Architecture

Multiple application programming interface (API) scripts were developed within version 15.6 of the Varian treatment planning system Eclipse Scripting API (ESAPI) to facilitate auto-planning. The framework for these scripts were derived from the results of our previously published works with VMAT TBI auto-planning (12, 13). The process was broken into two parts: preparation and optimization. By breaking the process into two distinct sections, the planner has a chance to review the generated tuning/optimization structures, the created plan(s), the isocenter and beam placement, and assigned optimization objectives prior to the optimization loop. Should a problem be discovered, the planner can easily fix the issue or rerun the preparation script rather than losing time optimizing plan(s) with a sub-optimal setup.

Once the auto-contours have been reviewed and the targets drawn and approved by the physician, the planner will launch the preparation script. The user will then select a plan template to use for the given patient, which will pre-populate all the relevant parameters for preparation. To guide the user where to go on the GUI, the tabs change color depending on the action that should be performed next: red indicates an action should be performed and green indicates the action on that tab is complete. Since generation of optimization/tuning structures and plan generation can take more than 30 seconds each (e.g., tuning structure generation can take up to 4 minutes depending on the complexity of the case and the resolution of the CT scan), asynchronous progress reporting was implemented for these operations so the user is aware of what the script is doing during these operations. Once the plan(s) have been prepared using the script, the user will save their changes, reload the patient in Eclipse, and review the output of the script.

Once the user is satisfied with the prepared plan(s), they will launch the optimization loop script, either from within Eclipse or on a thick-client workstation. The script will pick up where the preparation script left off and allow the user another chance to change the plan objectives and optimization constraints for this patient. Once the optimization loop is launched, it proceeds until all plan objectives are met or until the maximum number of user-requested iterations is reached. While the logic for the optimization loop was built on our previous code (13), several modifications were necessary to allow for sequential optimization of initial and boost CSI plans, specifically regarding plan evaluation. Since there is no available method within ESAPI v15.6 to generate a plan sum, a custom method was implemented to create a sum plan for evaluation of the plan objectives. The optimization loop script was designed to eliminate planner oversight/intervention so the user can launch the script at the end of the workday and return to plan(s) ready for review in the morning. Video demonstrations of both scripts are provided in the **Supplementary Material**.

The auto-planning code developed in this work has been made open-source under the MIT License via Github (https://github.com/esimiele/VMAT-TBI-CSI). In addition to the flexibility provided by the script GUIs, configuration and template files are provided with the scripts that are read at run-time. Users can simply adjust the configuration files and modify/add plan template files without having to adjust and recompile the underlying code, which allows users to adapt the scripts to their clinical environment and practice.

#### 2.2.2 Evaluation

The developed scripts were tested on two patient cohorts previously treated at our institution: ten patients treated to 12 Gy in 6 fractions and ten patients treated to 36 Gy in 20 fractions followed by an 18 Gy boost in 10 fractions. The 12 Gy cohort represents a simple case of auto-planning a single plan only, whereas the 36 Gy cohort represents the more challenging case of auto-planning two plans simultaneously where the achieved quality of one plan influences the achieved quality of the other and the quality of the composite sum.

The quality of auto-plans was compared to their clinical (i.e., manual) plan counterparts on the basis of clinically relevant DVH metrics such as target coverage, target hotspot, low isodose spill, etc. Paired t-tests were used to evaluate the significance of any observed differences where a p ≤ 0.05 was considered statistically significant. To further evaluate the quality of the auto-plans, two physicians and one physicist were asked to review the 40 plans in a blinded retrospective manner where all identifying information was removed from the plans. The reviewers were asked for each patient: 1) if each plan was clinically acceptable, and 2) between the two sets of plans, which would they choose for treatment and why. Finally, the auto- and manual-plans were compared based on required planning time. The time for manual planning was estimated based on time stamps in the Varian Aria system whereas the time for auto-planning was determined from evaluating the log files produced from the scripts.

### 2.3 Auto-plan checking

#### 2.3.1 Automated Plan Checker (APC)

The APC tool was adapted from a previously developed ESAPI script at our institution that focused on reducing treatment planning errors before they reached patient treatment (14). Liu et al. (14) utilized the *Six Sigma* DMAIC methodology combined with an FMEA analysis of our institution’s planning and treatment practice (14) to guide development of the APC. During testing and initial clinical implementation, they found APC to significantly reduce planning errors while simultaneously improving the efficiency of physics plan checking. This tool is still in use at our institution today and is routinely updated based on changes in workflow and planning practice. The present work built on the success of the APC and incorporated checks for identified failure modes during VMAT-CSI planning for both low-dose and high-dose CSI. The APC tool was modified and refined during the auto-planning development process and was thoroughly tested for false positives and negatives prior to clinical implementation.

#### 2.3.2 Robustness evaluation

To ensure the auto-plans are insensitive to small changes in longitudinal positions of the isocenters, plan robustness was tested for the 12-Gy cohort by shifting the isocenters of the upper spine and lower spine fields 3 mm in the superior direction. The change in the global max dose between the nominal and shifted plan was then compared between the manual and auto-plans.

## 3 Results

### 3.1 Auto-contouring

#### 3.1.1 Auto-contouring workflow

The auto-contouring model was deployed on the clinical workstation to read CT scans from a user-specified network location. The auto-planning script communicates with a DICOM Daemon to facilitate the delivery of planning CT scans to the network location, at which point auto-contouring would commence. The predicted mask for each organ-at-risk was transformed from a binary mask into an RT structure contour, and the output DICOM RT structure set file is identified by the auto-planning script and imported into the treatment planning system to be used for the subsequent planning procedure. The entire process takes less than 5 minutes.

#### 3.1.2 Auto-contouring evaluation

**Table 1** shows the auto-contour evaluation using Dice Similarity Coefficient (DSC), 95% Hausdorff distance (HD95), and a qualitative ranking by two physicians and one physicist (1 - acceptable, 2 - minor edits, 3 - major edits). The auto-contours achieved an average DSC of 0.71 ± 0.15, average HD95 of 4.81 ± 4.68, and average ranking of 1.22 ± 0.39 from the experts. The average ranking for all the structures was between 1 (acceptable) and 1.5. Optic chiasm was ranked with the worst score from the reviewers 1.45 ± 0.5 and the lowest DSC of 0.38 ± 0.21. On the other hand, brain received highest DSC of 0.97 ± 0.01. Reviewer 1 ranked 30 structures out of 500 (6%) as “3 - requiring major edits” with optic chiasm being most frequently ranked as “3” (4 out of 20 patients). Reviewer 2 ranked 16 structures out of 500 (3.2%) as “3 - requiring major edits” with optic chiasm (2 out of 20), pituitary (2 out of 20), cochlea R (2 out of 20) and cochlea L (2 out of 20) being most frequently ranked as “3”.

**Table 1:** Auto-contouring evaluation parameters: Dice Similarity Coefficient (DSC), 95% Hausdorff Distance (HD95), and a qualitative ranking by one physician and one physicist (1 acceptable, 2 minor edits, 3 major edits).

Structure	DSC	σ	HD95	σ	Reviewer Ranking	σ
Brain	0.97	0.01	2.64	1.93	1.20	0.30
BrainStem	0.76	0.12	4.11	1.88	1.10	0.25
Cochlea_L	0.52	0.29	5.27	11.66	1.30	0.74
Cochlea_R	0.51	0.28	5.39	12.06	1.30	0.74
Esophagus	0.66	0.12	10.75	15.39	1.25	0.34
Eye_L	0.86	0.20	1.85	0.57	1.00	0.00
Eye_R	0.87	0.20	1.83	0.62	1.00	0.00
Glnd_Submand_L	0.73	0.19	4.09	3.79	1.18	0.55
Glnd_Submand_R	0.75	0.16	3.31	2.16	1.10	0.32
Heart	0.91	0.03	4.81	2.23	1.40	0.35
Kidney_L	0.92	0.02	4.41	4.40	1.40	0.47
Kidney_R	0.92	0.04	4.16	4.90	1.28	0.48
Larynx	0.80	0.08	4.52	1.83	1.23	0.39
Lens_L	0.46	0.17	2.99	1.58	1.20	0.36
Lens_R	0.50	0.26	2.60	1.64	1.38	0.68
Lungs	0.96	0.02	3.64	7.31	1.33	0.38
OpticChiasm	0.38	0.21	5.76	3.49	1.45	0.50
OpticNrv_L	0.52	0.19	6.45	5.80	1.08	0.24
OpticNrv_R	0.59	0.19	5.24	4.37	1.13	0.40
OralCavity	0.88	0.06	3.64	1.29	1.13	0.23
Parotid_L	0.75	0.23	5.54	4.26	1.18	0.48
Parotid_R	0.77	0.20	5.25	2.69	1.10	0.32
Pituitary	0.47	0.24	2.98	1.79	1.33	0.74
SpinalCord	0.74	0.07	14.85	17.80	1.08	0.18
Thyroid	0.68	0.09	4.12	1.47	1.35	0.32
Average	0.71	0.15	4.81	4.68	1.22	0.39

Overall agreement rate between reviewers for all the structure ranking was 80.8%. For two patients with unusual anatomy (patient with missing anterior skull after surgery, and patient with hardware and hyper-flexed neck), the auto-segmentation performed the worst where 10 out of 25 structures required major editing based on Reviewer 1 ranking. Overall, 60% of structures had DSC ≥0.7 and reviewer ranking ≤1.4.

### 3.2 Auto-planning

**Figure 1:**
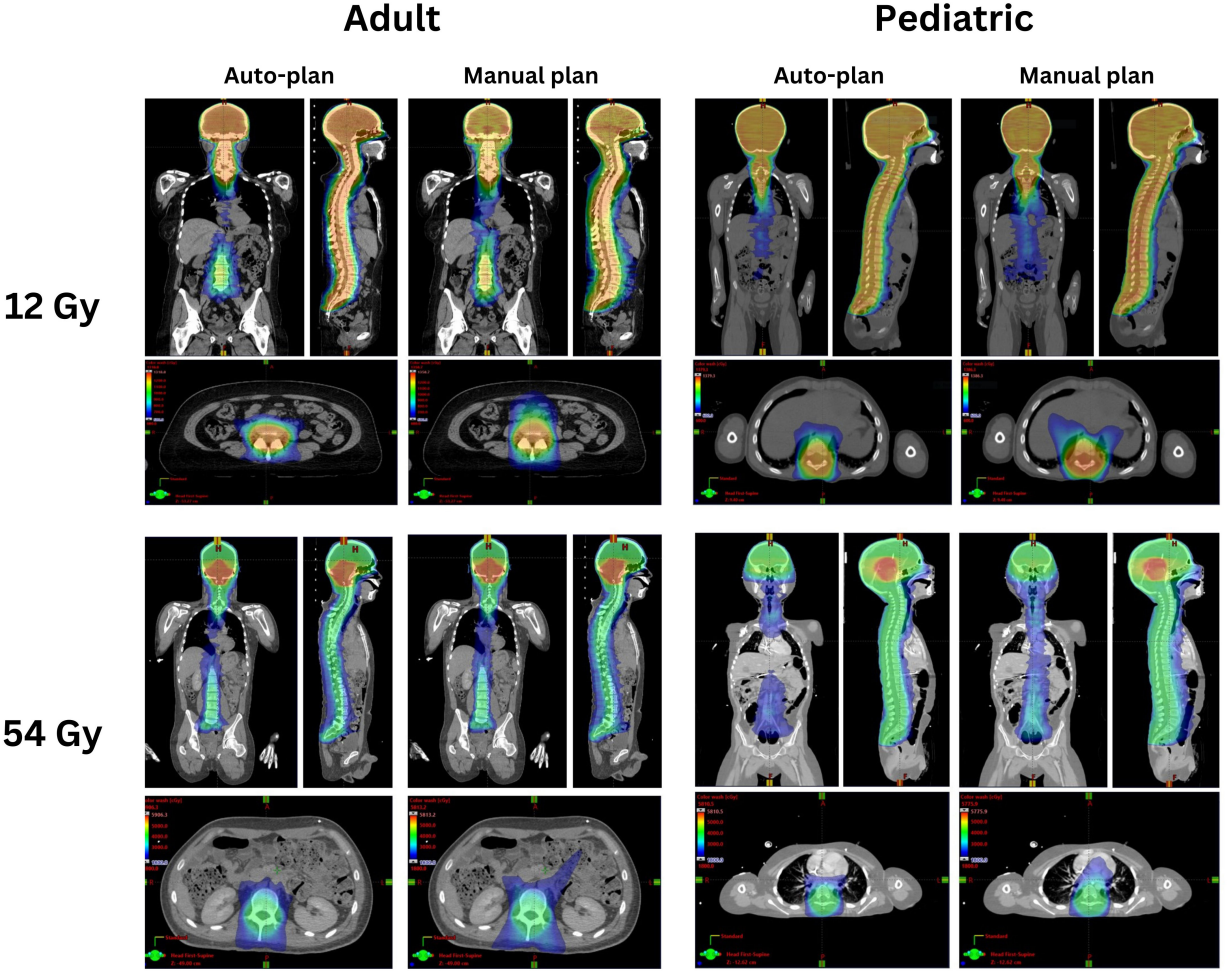
Comparison of the auto-plans and manual plans for one adult and one pediatric patient for 12 Gy and 54 Gy dose regimens. The dose clouds have been thresholded to 50% of the initial plan prescription dose.

**Figure 1** shows the manual and auto plan dose distributions for a pediatric patient and an adult patient for the low-dose and high-dose regimens of VMAT-CSI. The dose cloud is thresholded to 50% of the initial plan prescription dose. As seen from the axial, coronal, and sagittal slices, the auto plan exhibits less anterior dose spillage compared to the manual plan.

**Figure 2:**
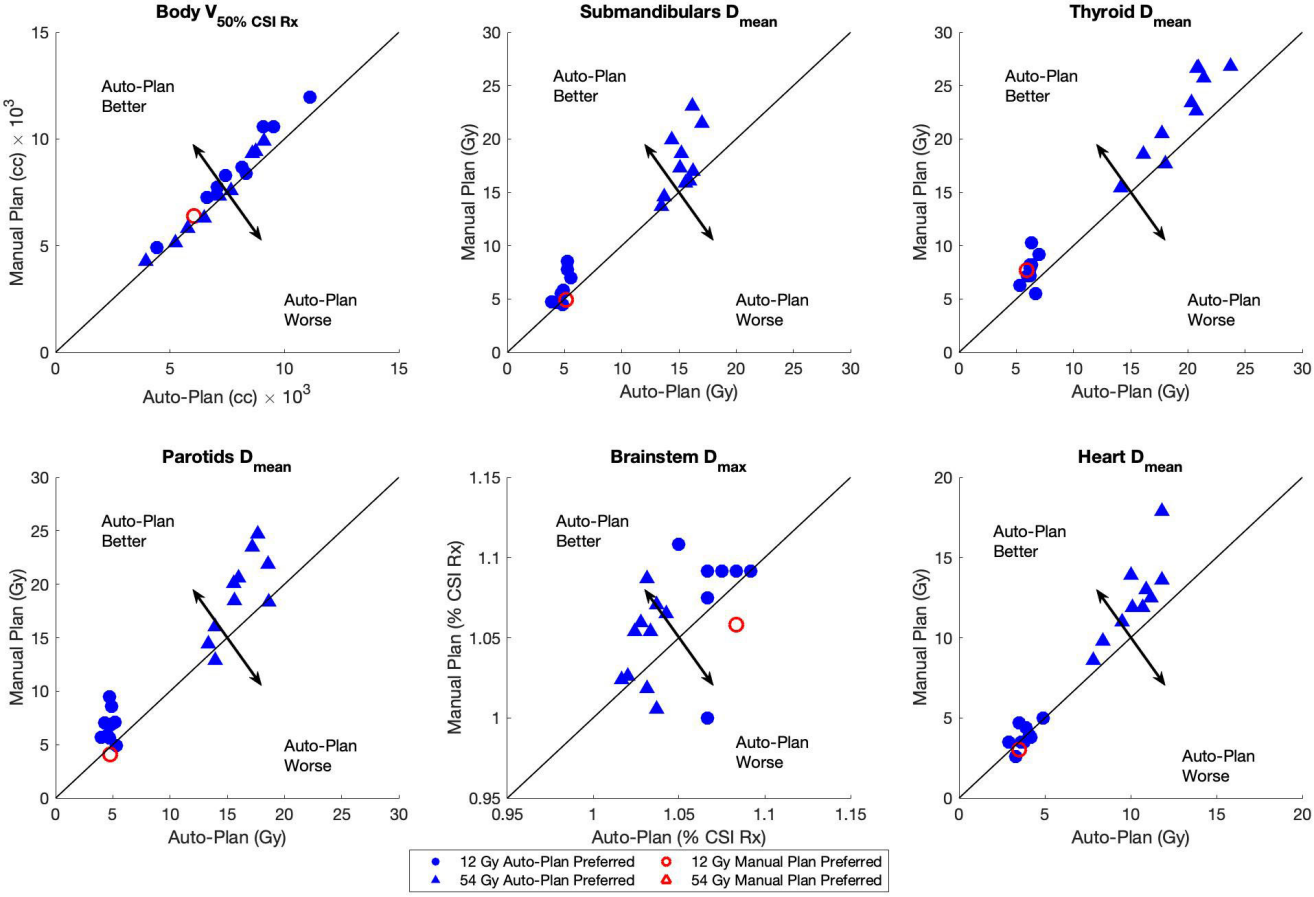
Dosimetric scatterplots of the six most statistically significant quantities between the manual plans and auto-plans for 12 Gy (circles) and 54 Gy (triangles) dose regimens: body V50%, and Dmean for submandibular glands, parotids, thyroid, and heart and Dmax for brainstem. The Brainstem Dmax was normalized to the CSI Rx dose. The plan preference was based on the majority consensus response (from at least 2 reviewers). The black arrows in each plot indicate the direction of auto-plan superiority/inferiority relative to the line of plan equality.

Dosimetric scatterplots of the six most statistically significant quantities between the manual and auto-plans for the low-dose and high-dose regimens of VMAT-CSI are illustrated in **Figure 2**. The low-dose regimen is represented by circles while the high-dose regimen is represented by triangles. A clear preference by the medical experts toward the auto-plans is seen by the color shading of the shapes.

The average achieved dosimetric indices for the auto- and clinical manual-plans are shown in **Tables 2** and **3** for the 12 Gy cohort and 36 Gy initial with 18 Gy boost cohort, respectively. The majority of the observed differences were not statistically significant with some notable exceptions: intermediate-dose spill (body V50%), parotids D_mean_, submandibulars D_mean_, and thyroid D_mean_ were significantly lower for the auto-plans as compared to the manual plans for both cohorts. In addition, brainstem D_max_ (constraint of D_max_ *<*55 Gy) was significantly lower for the auto-plans compared to the manual plans for the 36 Gy cohort (**Table 3**).

**Table 2:** Average dosimetric indices and their associated p-values for the auto- and manual plans for 12 Gy CSI.

Dosimetric Parameter	Constraint	Auto-planAverage	σ	Manual Plan Average	σ	% diff	p-value
**PTV CSI 12 Gy Dmax** %	**< 115 %**	**112.5 %**	**0.2 %**	**113.7 %**	**1.8 %**	**-1.1 %**	**02**
**PTV CSI 12 Gy D0.03cc** %	**< 113 %**	**111.7 %**	**0.2 %**	**113.1 %**	**1.9 %**	**-1.2 %**	**02**
PTV CSI 12 Gy V110 %	< 1	0.1 %	0.1 %	2.6 %	5.9 %	-97.9 %	10
**Body V6Gy (cc)**		**7772.1**	**1906.1**	**8480.5**	**2107.2**	**-8.3 %**	**0001**
BrainStem Dmax (Gy)	<13.2 Gy	12.9	0.1	13.0	0.5	-1.1 %	20
Cochleas Dmean (Gy)	<12.5 Gy	12.4	0.2	12.4	0.4	0.0 %	31
Esophagus Dmean (Gy)	<12.0 Gy	8.3	0.9	8.6	0.9	-3.3 %	09
**Glnd_Submands Dmean (Gy)**	**<5.5 Gy**	**4.9**	**0.5**	**5.9**	**1.4**	**-17.6 %**	**01**
Eyes Dmax (Gy)	<12.5 Gy	12.5	0.7	12.8	0.9	-2.4 %	06
Eyes Dmean (Gy)	<12.0 Gy	10.3	0.9	9.7	1.5	6.7 %	06
Heart Dmean (Gy)	<4.5 Gy	3.8	0.6	3.8	0.7	-0.5 %	46
Kidneys Dmean (Gy)	<3.0 Gy	3.0	0.3	3.1	1.0	-3.0 %	38
**Larynx Dmean (Gy)**	**<8.0 Gy**	**6.4**	**0.7**	**7.2**	**1.2**	**-10.6 %**	**02**
Lenses Dmax (Gy)	<10.0 Gy	9.4	1.3	8.1	2.3	16.8 %	06
Lungs Dmean (Gy)	<3.0 Gy	3.2	0.3	3.4	0.8	-7.4 %	13
OpticChiasm Dmax (Gy)	<13.2 Gy	12.7	0.2	12.8	0.5	-0.9 %	23
Optic Nerves Dmax (Gy)	<13.2 Gy	12.8	0.1	12.9	0.5	-0.6 %	32
OralCavity Dmean (Gy)	<3.5 Gy	3.4	0.3	3.7	1.4	-7.9 %	27
**Parotids Dmean (Gy)**	**<4.5 Gy**	**4.7**	**0.4**	**6.5**	**1.6**	**-27.6 %**	**004**
Pituitary Dmax (Gy)	<13.2 Gy	12.7	0.2	12.7	0.4	-0.3 %	36
SpinalCord Dmax (Gy)	<13.2 Gy	13.2	0.2	13.2	0.6	0.1 %	48
**Thyroid Dmean (Gy)**	**<6.5 Gy**	**6.2**	**0.5**	**7.8**	**1.4**	**-20.1 %**	**002**

All the plans were normalized to PTV CSI D95%=12Gy. A p-value ≤0.05 was considered statistically significant (bold text).

**Table 3:** Average dosimetric indices and their associated p-values for the auto- and manual plans for the CSI plan sum (36 Gy initial plan + 18 Gy boost).

Dosimetric Parameter	Constraint	Auto-plan Average	σ	Manual Plan Average	σ	% diff	p-value
PTV Boost 54Gy D95 %	≥ 100 %	100.3 %	0.7 %	100.7 %	1.3 %	-0.4 %	0.20
PTV Boost 54Gy Dmax %	< 110 %	107.9 %	1.1 %	107.2 %	1.2 %	0.7 %	0.07
PTV Boost 54Gy D0.03cc %	< 110 %	107.6 %	1.0 %	106.7 %	1.3 %	0.8 %	0.06
PTV CSI 36Gy D95	≥ 100 %	102.1 %	0.5 %	100.9 %	0.3 %	1.2 %	0.06
**Body V18Gy (cc)**		**6966.9**	**1658.8**	**7244.8**	**1903.1**	**-3.8 %**	**0.02**
**BrainStem Dmax (Gy)**	** < 55 Gy**	**55.6**	**0.4**	**56.5**	**1.4**	**-1.5 %**	**0.04**
Cochleas Dmean (Gy)	< 45 Gy	42.1	2.5	41.2	4.0	2.2 %	0.18
**Esophagus Dmean (Gy)**	** < 30 Gy**	**25.0**	**3.9**	**26.3**	**3.9**	**-4.9 %**	**0.01**
Eyes Dmax (Gy)	< 45 Gy	41.1	3.5	42.1	3.6	-2.4 %	0.10
Eyes Dmean (Gy)	< 35 Gy	28.3	5.7	30.2	6.4	-6.4 %	0.09
**Glnd_Submands Dmean (Gy)**	** < 20 Gy**	**15.3**	**1.1**	**17.8**	**3.0**	**-14.1 %**	**0.01**
**Heart Dmean (Gy)**	** < 10 Gy**	**10.2**	**1.3**	**12.4**	**2.5**	**-17.6 %**	**0.001**
Kidneys Dmean (Gy)	< 10 Gy	10.4	1.5	9.6	1.9	8.1 %	0.07
Larynx Dmean (Gy)	< 25 Gy	21.0	2.9	21.3	4.8	-1.2 %	0.38
Lenses Dmax (Gy)	< 15. Gy	20.6	7.2	23.2	9.5	-11.1 %	0.17
Lungs Dmean (Gy)	< 10 Gy	9.7	3.7	9.7	3.4	0.4 %	0.46
OpticChiasm Dmax (Gy)	< 55 Gy	51.2	4.4	50.9	4.1	0.4 %	0.31
OpticNerves Dmax (Gy)	< 55 Gy	47.4	6.0	47.2	5.7	0.3 %	0.35
OralCavity Dmean (Gy)	< 15 Gy	14.5	1.6	14.8	3.5	-1.8 %	0.37
**Parotids Dmean (Gy)**	** < 15 Gy**	**16.1**	**1.9**	**19.1**	**3.8**	**-15.9 %**	**0.003**
Pituitary Dmax (Gy)	< 60 Gy	52.5	4.8	52.4	4.6	0.1 %	0.43
SpinalCord Dmax (Gy)	< 55 Gy	46.3	9.1	46.2	8.9	0.3 %	0.24
**Thyroid Dmean (Gy)**	** < 20 Gy**	**19.4**	**2.8**	**22.4**	**4.2**	**-13.5 %**	**0.0004**
Ovaries Dmax (Gy)	< 10 Gy	9.1	n/a	18.5	n/a	-51.1 %	n/a
Ovaries Dmean (Gy)	< 5 Gy	3.3	n/a	3.3	n/a	-1.5 %	n/a

A p-value ≤0.05 was considered statistically significant (bold text).

**Figure 3:**
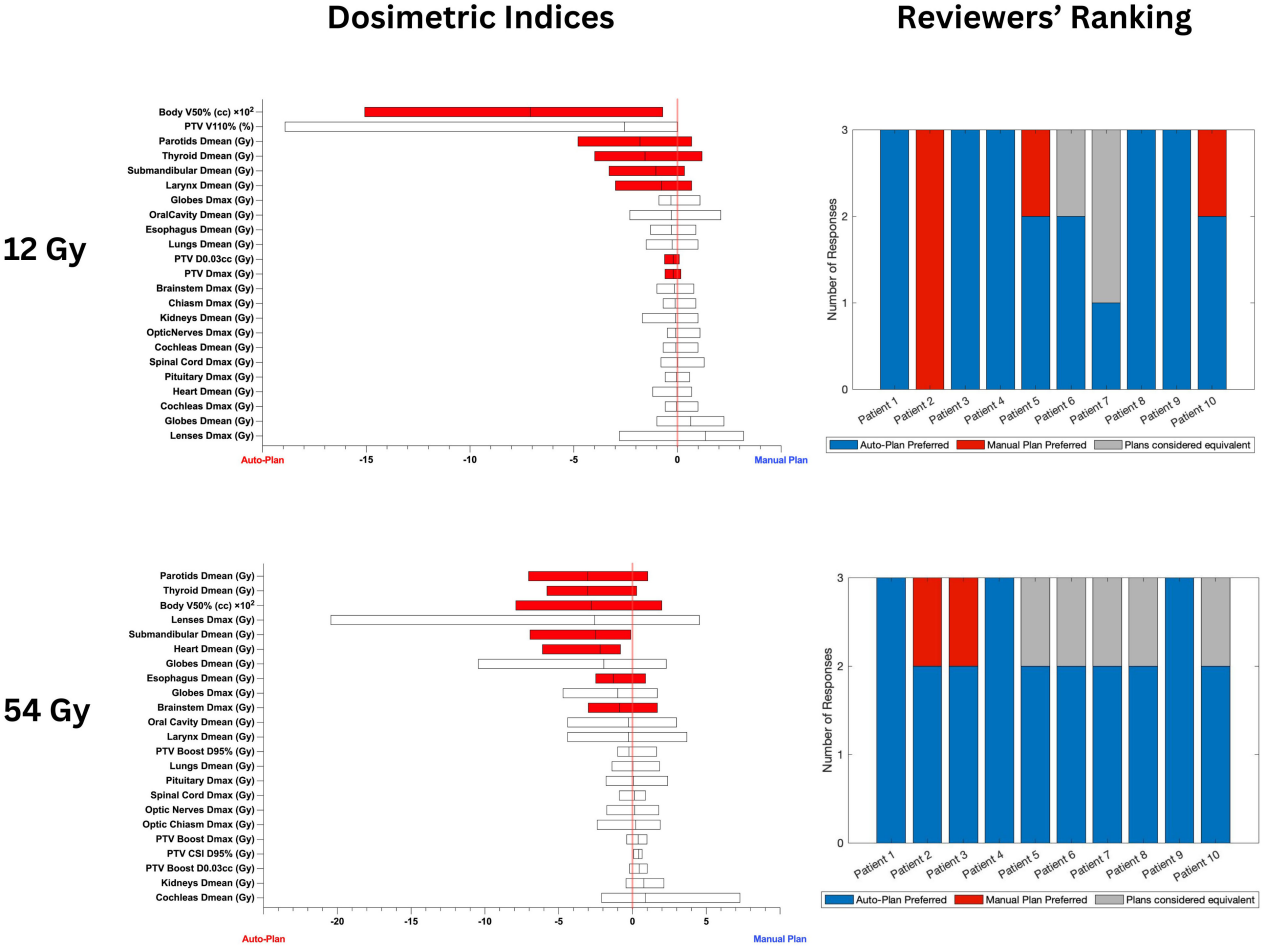
Box plots on the left show the differences in achieved plan quality metrics between the auto-plans and manual plans for 12 Gy and 54 Gy CSI cohorts. Parameters plotted in the left part of the graph show auto-plan superiority. Red shading denotes statistically significant differences. The differences in PTV coverage and plan heterogeneity were not statistically significant. Plots on the right show the blinded plan review results by 3 experts: blue denotes auto-plan preference, gray denotes auto- and manual plan equivalency and red shows manual plan preference.

**Figure 3** shows the box plots of differences in achieved plan quality metrics (left) and reviewers’ ranking of the plans (right) indicating auto-plan preference 88.3% of the time.

### 3.3 Auto-plan checking

#### 3.3.1 Automated Plan Checker (APC)

A sample plan check report generated by the APC is available in the **Supplementary Material**. Information regarding the patient, course, and treatment plan sum name is displayed in the header of the report. The item checks of the VMAT-CSI plan sum are shown in the left column. The middle column reports the status of the item check. The right column provides a description for the result of the check. In addition to the routine checks e.g., prescription, dose, dose rate, energy, etc, the APC tool checks the geometry of the CSI beams, location of isocenters and verifies the entered shifts in the Aria dynamic document used by the therapists during treatment delivery.

#### 3.3.2 Robustness evaluation

When evaluating robustness for all the plans by deliberately shifting isocenters into each other by 3 mm, the median global D_max_ increase was lower with auto-plans compared to manual plans (5.9% vs 9.6%, p = 0.037). The standard deviation of global D_max_ increase was also smaller for auto-plans compared to manual plans (4.3% vs 8.3%) indicating a more consistent change in plan heterogeneity with isocenter positioning uncertainty with the auto-plans as compared to the manual plans.

## 4 Discussion

Patients requiring CSI treatments can include pediatric, adolescent, and adult patient populations. Therefore, any automated planning tool would need to be applicable to a large range of patient, organ, and target sizes. The primary challenge in developing accurate deep learning auto-contouring models for pediatric patients is the greater variation in organ structure size and shape as compared to adult patients (15, 16).

Efforts have been made to develop robust auto contouring tools to alleviate the demands of pediatric treatment planning and remove inter-planner variability while improving planning time efficiency and plan accuracy. Qiu et al. (17) constructed and evaluated a Triplet-Attention U-Net (TAU-Net) auto-contouring model to contour important pediatric skeletal growth centers in the craniofacial, shoulder, and pelvic regions with the objectives of mitigating growth abnormalities induced by radiation treatment. Hernandez et al. (9) automated the end-to-end treatment planning process for 3D CSI to provide a tool for resource-constrained communities. Auto-contouring in their work was performed with a CNN model for HN organ structures and a nn-UNet model for the remaining structures required for 3D CSI. Adamson et al. (18) developed and evaluated a fully convolutional network for pediatric CT organ delineation which proved to be generalizable across CT scanner model protocols and patient age. Similar to the approach by Hernandez et al. (9), auto-contouring in this work was achieved by utilizing a combination of two models to achieve satisfactory contours: a 2.5D UNet model for contouring small HN structures (e.g., optic nerves, chiasm, etc.) and 2D UNet model for contouring the remaining OARs. This hybrid approached provided better flexibility and accuracy in generating accurate contours for very small structures (e.g., chiasm, optic nerves, etc.) and structures of varying size depending on patient size and age (e.g., lungs, kidneys, etc.).

Our group previously developed an automated planning process for VMAT-TBI (12, 13), which allowed us to leverage the framework from that study in the present work. Similar to our previous works (12, 13), multiple API scripts were developed to streamline the auto-planning process. Following the initial introduction of VMAT-CSI (7) into our clinical practice, planning for these cases consisted of using clinical protocol templates, which contained the necessary target and OAR structures, beam arrangement, optimization objectives, and plan information. While these templates are helpful, they have several limitations, the greatest of which is they are not patient-specific, which is critical for cases where the target shape and size can significantly vary. Thus, the planner would be required to modify the initial beam and plan arrangement to conform to the patient-specific anatomy. Furthermore, the template only contains the necessary OAR structures, but not any of the tuning/optimization structures, which must be generated manually by the planner. Finally, due to the large calculation volume, the memory requirements of optimization and dose calculation significantly slow down the planning process, which reduces the number of tasks a planner could work on simultaneously.

The developed preparation script can generate optimization structures, determine the required number and location of isocenters, generate the plan, place the appropriate number of beams, and assign optimization constraints in 2 minutes on average and 5 minutes maximum. Combined with the developed auto-contouring model, contouring normal tissues and preparing the patient plan for optimization can be achieved in less than 15 minutes as compared to several hours when done manually. The optimization loop script permitted autonomous optimization of the CSI plan(s) without requiring planner intervention.

The developed APC for VMAT-CSI allows the physicist performing the second check to readily review items that may be detrimental to patient safety during treatment. Of the items that are checked, verification of the dosimetric shifts between isocenters is particularly important for VMAT-CSI as without this information, the therapists will be missing important pieces of treatment delivery instructions. This may necessitate last-minute shift calculations, searching the treatment plan report documentation, etc., which can be an error-prone process when under time pressure. In the example provided in the **Supplementary Material**, the APC recognized that the dosimetric shifts document was not found and flagged it for review by the second checking physicist.

Significant improvements in multiple clinically relevant metrics were observed for both sets of patient cohorts considered in this study (12 Gy only and 36 Gy initial with 18 Gy boost) for the auto-plans compared to the manual plans (**Tables 2**, **3**). Due to these improvements, the auto-plans were preferred by majority of medical experts for 18 out of the 20 patients selected for this study (90%) as highlighted in **Figure 3**. In the two remaining cases, the majority of reviewers assigned them as equivalent (n=1) and manual plan preferred (n=1). For patient 2 in the 12 Gy cohort all reviewers preferred the manual plan as the dose to the parotids, globes, and lenses were slightly lower with the manual plan compared with the auto-plan. However, the auto-plan dosimetric indices for these structures were still below the acceptable threshold. All 20 plans generated by the auto-planning scripts were deemed clinically acceptable by all 3 reviewers. Of particular concern with CSI treatments is intermediate-dose spill, which could result in secondary malignancies later in life due to the large target volume. To ensure tight conformity of the prescription dose to the target, the developed auto-planning scripts prioritize body V50% using a ring structure of 2 cm thickness and 1.5 cm margin from the target structure. Compared to the manual plans, the auto-plans achieved significant reductions in body V50% without sacrificing target coverage or heterogeneity.

While substantial advancements were made toward automating VMAT CSI treatment planning in this work, there were also several limitations to the present study. First, the size of both the low-dose (Rx dose = 12 Gy) and high-dose (36 Gy initial with 18 Gy boost) patient cohorts was relatively small at 10 patients per dose regimen. Second, the number of expert reviewers was relatively limited at three, which may skew the blinded plan review results. While the sample size of each CSI dose cohort was relatively limited for this work, feedback mechanisms from dosimetry have been implemented into our clinical practice to improve the accuracy and robustness of the automated planning scripts as more patients are treated with this technique. Third, the developed auto-planning scripts were built using version 15.6 of Eclipse and all API functions and calls were targeted at this version of ESAPI. There is no guarantee the scripts will work for any other version of Eclipse, which may limit widespread adoption of the scripts at other institutions. Future work includes expanding the number of patients for each dosing cohort to better understand the dosimetric differences between the manual and auto-plans. However, the described process and autoplanning software has been clinically implemented in the authors institution given the positive initial results from this work regarding efficiency and standardization improvements in VMAT-CSI treatment planning without comprosing plan quality.

## 5 Conclusions

Significant reductions in contouring and planning time were achieved without sacrificing plan quality using the developed auto-planning process. The required time for contouring and planning decreased from 5–6 hours (manual planning) to consistently between 1–2 hours (auto-planning). Furthermore, statistically significant improvements were achieved in OAR sparing using the developed scripts. The auto-plans were marked as equivalent or superior to the manual plans 88.3% of the time from the blinded review by the three experts. The scripts and documentation are publicly available on GitHub (https://github.com/esimiele/VMAT-TBI-CSI) so other institutions can adopt the developed auto-planning process into their own practice.

## Data availability statement

The raw data supporting the conclusions of this article will be made available by the authors, without undue reservation.

## Ethics statement

Ethical approval was not required for the study involving humans in accordance with the local legislation and institutional requirements. Written informed consent to participate in this study was not required from the participants or the participants’ legal guardians/next of kin in accordance with the national legislation and the institutional requirements.

## Author contributions

ES: Conceptualization, Data curation, Formal analysis, Funding acquisition, Investigation, Methodology, Project administration, Resources, Software, Supervision, Validation, Visualization, Writing – original draft, Writing – review & editing. IR: Conceptualization, Data curation, Formal analysis, Funding acquisition, Investigation, Methodology, Project administration, Resources, Software, Supervision, Validation, Visualization, Writing – original draft, Writing – review & editing. J-YW: Conceptualization, Data curation, Formal analysis, Funding acquisition, Investigation, Methodology, Project administration, Resources, Software, Supervision, Validation, Visualization, Writing – original draft, Writing – review & editing. YC: Writing – review & editing, Data curation, Methodology, Software. YL: Writing – review & editing, Data curation, Methodology. YS: Writing – review & editing, Data curation, Methodology. LS: Writing – review & editing, Methodology, Resources. YY: Methodology, Writing – review & editing. LX: Writing – review & editing, Methodology, Resources, Supervision. IG: Data curation, Investigation, Writing – review & editing. SH: Methodology, Project administration, Resources, Software, Supervision, Validation, Visualization, Writing – original draft, Writing – review & editing, Conceptualization, Data curation, Formal analysis, Funding acquisition, Investigation. NK: Conceptualization, Data curation, Formal analysis, Funding acquisition, Investigation, Methodology, Project administration, Resources, Software, Supervision, Validation, Visualization, Writing – original draft, Writing – review & editing.
