# Supplementary figures and images for: Automated contouring, treatment planning, and quality assurance for VMAT craniospinal irradiation (VMAT-CSI)
Source: Front Oncol. 2024 Apr 10;14:1378449. doi: 10.3389/fonc.2024.1378449 (PMC11039907; doi:10.3389/fonc.2024.1378449)

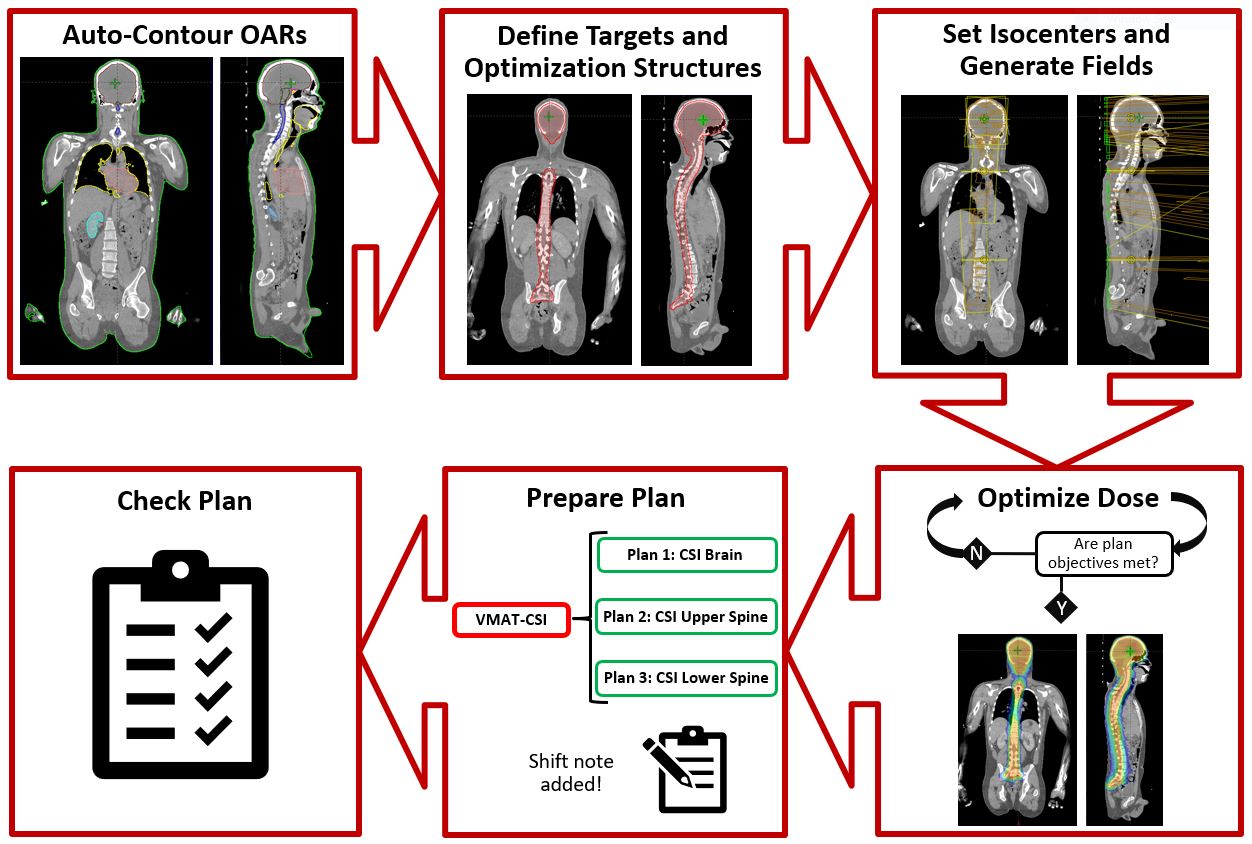

Supplement: Supplementary file 1 [file Image_1.jpeg]

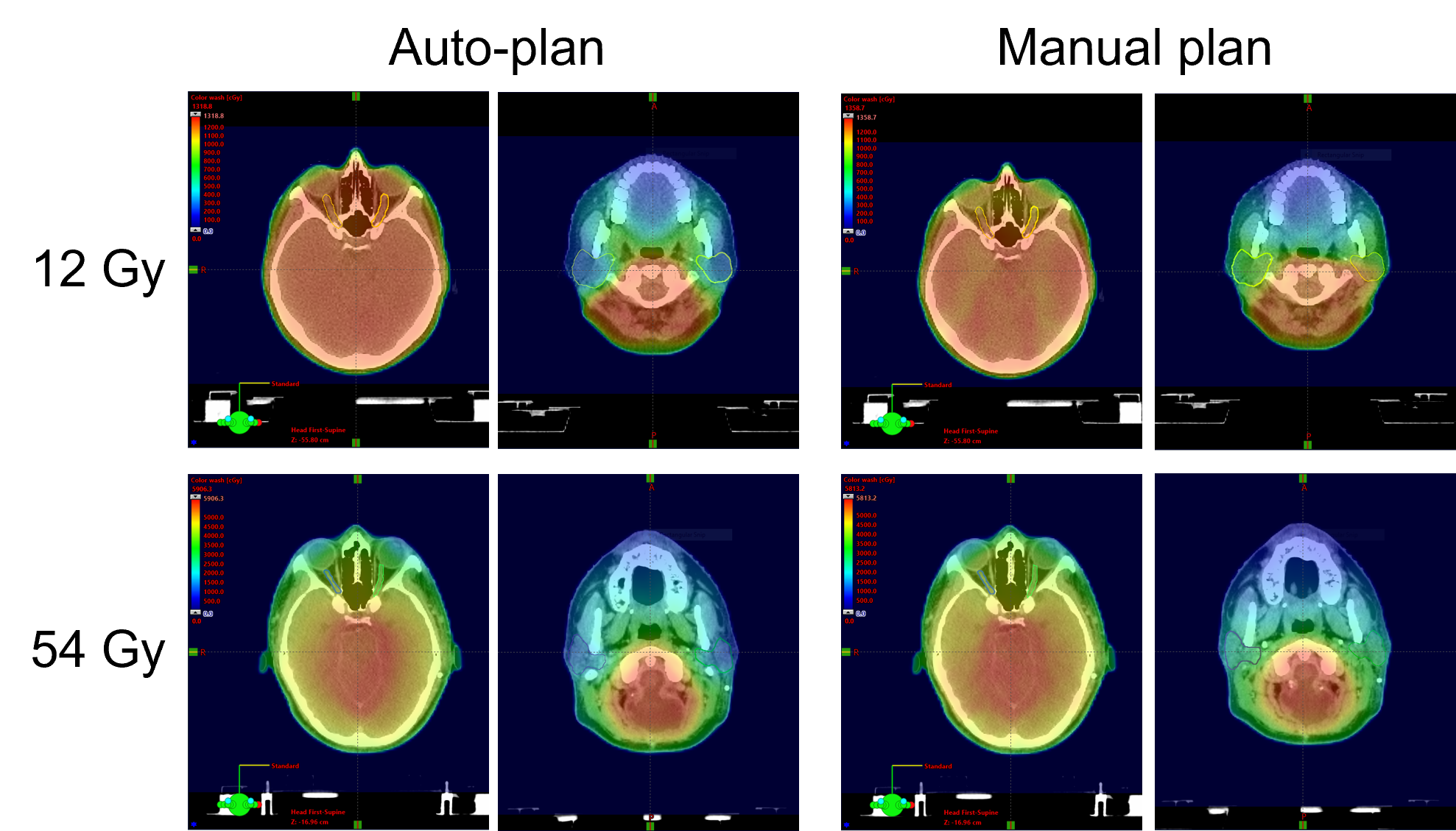

Supplement: Supplementary file 2 [file Image_2.png]

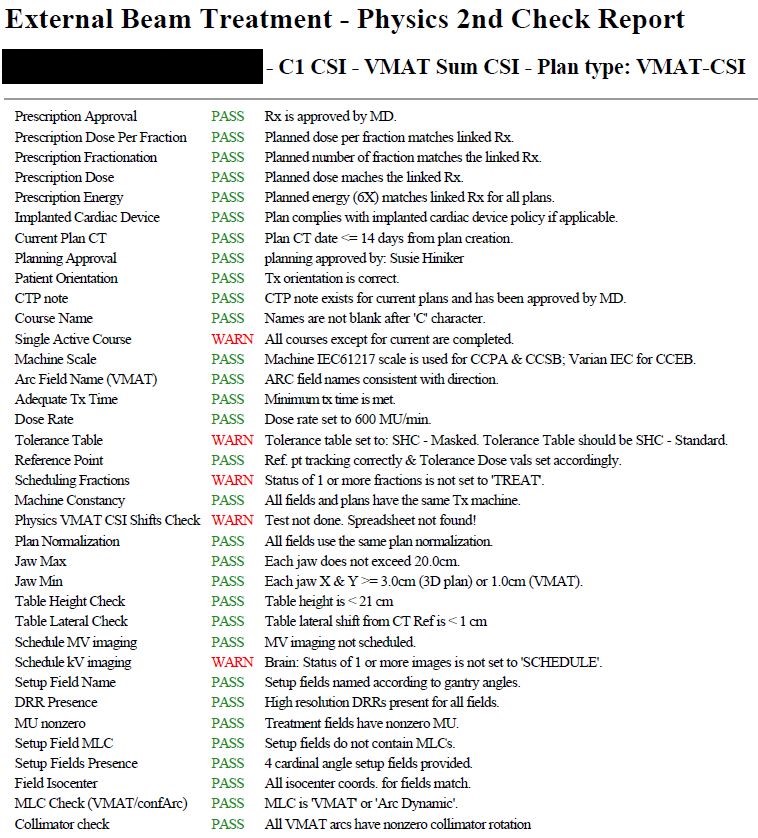

Supplement: Supplementary file 3 [file Image_3.jpg]
